# Quasi-simultaneous multiplane calcium imaging of neuronal circuits

**DOI:** 10.1364/BOE.10.000267

**Published:** 2018-12-19

**Authors:** Ee Zhuan Chong, Mariangela Panniello, Inês Barreiros, Michael M. Kohl, Martin J. Booth

**Affiliations:** 1 Department of Engineering Science, University of Oxford, Parks Road, OX1 3PJ, UK; 2 Department of Physiology, Anatomy and Genetics, University of Oxford, Parks Road, OX1 3PT, UK; 3 michael.kohl@dpag.ox.ac.uk; 4 martin.booth@eng.ox.ac.uk

## Abstract

Two-photon excitation fluorescence microscopy is widely used to study the activity of neuronal circuits. However, the fast imaging is typically constrained to a single lateral plane for a standard microscope design. Given that cortical neuronal networks in a mouse brain are complex three-dimensional structures organised in six histologically defined layers which extend over many hundreds of micrometres, there is a strong demand for microscope systems that can record neuronal signalling in volumes. Henceforth, we developed a quasi-simultaneous multiplane imaging technique combining an acousto-optic deflector and static remote focusing to provide fast imaging of neurons from different axial positions inside the cortical layers without the need for mechanical disturbance of either the objective lens or the specimen. The hardware and the software are easily adaptable to existing two-photon microscopes. Here, we demonstrated that our imaging method can record, at high speed and high image contrast, the calcium dynamics of neurons in two different imaging planes separated axially with the in-focus and the refocused planes 120 µm and 250 µm below the brain surface respectively.

## 1. Introduction

With the advancement of ultrafast laser technology, two-photon excitation fluorescence (2PEF) microscopy is becoming a common imaging tool in life science research. In particular, 2PEF microscopy is well suited for studying the neuronal activity in awake, behaving animal models. Cortical circuits process information in a highly distributed, parallel manner in three-dimensional (3D) space distributed over six anatomically distinct layers. Precisely how neural activity in these different layers enables information processing remains a key question in the field of neuroscience. Neural network models highlight the importance of quantitative understanding of the relationship between the activities in the input and the output layers on a trial-to-trial, rather than on an averaged basis [1,2]. In view of that, various techniques have been developed to enable fast 3D scanning in multiphoton microscopy. For example, several groups have developed versatile 3D random excitation for functional neural imaging with ultrafast acquisition rate based on the free beam steering capability afforded by pairing acousto-optic deflectors (AODs) [3–5]. Electrically tunable lens (ETL) is another useful device to aid the axial optical scanning without direct mechanical interference on the specimen. Because of the ease of use, ETL appeals to many scientists in developing new imaging techniques. Its applications cover not only the widefield and the confocal microscopy fronts [6,7] but also extend further to enhance multiphoton imaging techniques enabling the rapid excitation within volumes [8–11]. The remote focusing technique can assist high axial scan rates in a high numerical aperture (NA) condition without introducing aberrations [12–14]. This is particularly favourable for *in vivo* optical imaging in which: (1) high photon flux at the focus is crucial for the non-linear fluorescence excitation; (2) mechanical agitation of the specimen must be avoided. Apart from that, wavefront shaping devices such as spatial light modulators (SLM) and deformable mirrors (DM) have played important roles in brain imaging. For instance, their applications can be found not only in the aberration correction for *in vivo* imaging [15,16] but also in the multi-site targeted excitation of neurons [17,18] and the rapid spatiotemporal remote focusing [19]. Also, in conjunction with the DM based axial refocusing, concurrent multifocal detection can be realised through multiplexing the laser pulse train [20,21]. Similar time multiplexing via pulse delay but with added spatial beam separation through multiple beamsplitters has been reported to enable simultaneous multiplane 2P calcium imaging [22]. While the aforementioned techniques have various technical merits, their complexity hampers easy incorporation into existing multiphoton microscopy systems.

In order to capture the fluorescence of genetically encoded calcium indicators GCaMP [23] from axially separated neurons, we set out to develop an imaging technique that overcomes the slow axial scanning in a standard multiphoton microscope while retaining the planar image quality at minimal cost. This was achieved based on the idea of creating instantaneous optical switching of the laser propagation towards different beam pathways constructed with and without the remote focusing system. Thus, an AOD was integrated into the multiphoton microscopy system to obtain fast, inertia-free optical path switching. This permitted beam steering within microseconds to access different optical pathways that were configured to refocus the beam. One beam path entered the microscope in the conventional manner, whereas the other beam path passed through a remote focusing system that could translate the focal plane by up to few hundreds of micrometres away from the nominal focal plane. In attaining a high NA refocusing over an extended volume for an existing commercial microscope, we adopted the remote focusing technique by incorporating a pair of high NA lenses outside the microscope body with one lens allowed to translate in the z direction. This technique allows the axial translation of the focal spot along the optical axis without introducing substantial aberrations and circumvents the mechanical interference on both the animal specimen and the focusing objective lens. Integration of both the AOD switching and the remote focusing techniques can be easily implemented to convert a standard single plane scanning microscope into a fast switching multiplane microscopy system for studying stimulated responses in a 3D neuronal circuit at a fraction of the cost of more complex multiplane imaging methods.

Here, we characterised the performance of this system using a 3D bead sample and demonstrated its application through multiplane recording of neocortical circuit activity with neurons expressing GCaMP6s.

## 2. Design principles and validation

### 2.1 Quasi-simultaneous multiplane imaging system design

#### 2.1.1 Acousto-optic deflector for fast switching and its dispersion compensation

**Fig. 1:**
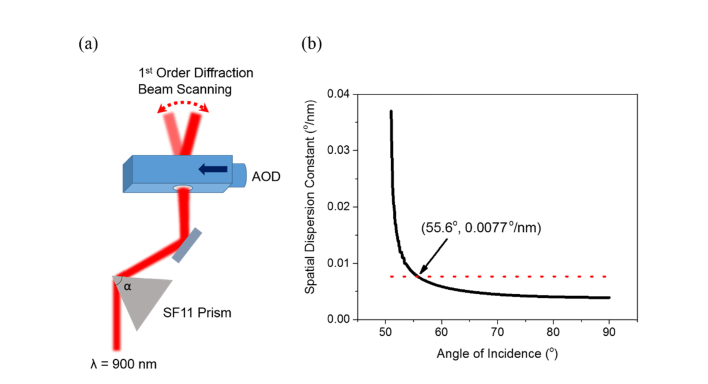
Pre-compensation of spatial dispersion in AOD using SF11 prism. (a) Schematic of pairing prism-AOD configuration for AOD dispersion compensation. The arrow on the AOD depicts the propagation of acoustic wave and α is the apex angle of SF11 prism *i.e.* 60°. (b) Calculated spatial dispersion constant of SF11 prism as a function of incident angle of 900 nm laser. The red dotted line is the intrinsic spatial dispersion constant of AOD acting as 1D scanner. The intersection point is the compensation instance where both dispersion constants cancel and the corresponding angle of incidence of the laser is 55.6°.

An AOD affords a motion-free laser beam steering capability. However, the diffractive nature of the tellurium dioxide (TeO_2_) crystal that constitutes the main driving component of the AOD is wavelength sensitive. Depending on the spectral bandwidth of the laser source used, the beam may experience appreciable spatial and temporal dispersion propagating through the AOD. In most cases in multiphoton microscopy, where a Ti:Sapphire ultrafast laser with the spectral bandwidth of ~10 nm is the preferred excitation source, the beam will be stretched spatio-temporally by the AOD leading to a significant reduction in the 2P excitation power and adverse distortion in the acquired fluorescence images. Therefore, pre-compensation for the AOD spatio-temporal dispersion has to be taken into account. Here, we used a SF11 equilateral dispersive prism (PS859, Thorlabs, USA) to recover the initial laser beam shape and its pulse duration [24–27]. Because the AOD functions as the fast horizontal beam scanner in the quasi-simultaneous multiplane imaging system, the prism was placed before the AOD so that the incident beam at the prism remained stationary introducing the negative group delay dispersion (GDD) required to cancel the positive dispersion induced in the AOD (see Fig. 1(a)

). However, to compensate for the dispersion utilising a prism, the procedures of positioning the prism can be divided into two stages. The first stage is to determine the correct incident angle of the laser at the prism to compress the distorted beam shape, while the second stage is to determine the correct separation between the prism and the AOD to minimize the broadened pulse duration.

To function in the Bragg regime as an optical scanner, the first order diffraction angle of AOD is defined as [28]

$$\theta_{1st}=\frac{\lambda f}{V}$$ (1)

where *θ*_1st_ is the first order diffraction angle, *λ* is the laser wavelength, *f* is the carrier frequency of the acoustic wave and *V* is the velocity of the acoustic wave. From Eq. (1), we can derive the spatial dispersion constant, *σ_AOD_* to be

$$\sigma_{AOD}=\frac{d\theta_{1st}}{d\lambda }=\frac{f}{V}$$ (2)

For the AOD model chosen (DTSX-400-980, AA Optoelectronic, France) in developing the multiplane functional imaging system, the corresponding central carrier frequency, *F_c_* at the laser wavelength 900 nm is 87.5 MHz and the acoustic velocity, *V* is 650 ms^−1^. All analyses for the spatial and temporal dispersion compensation were conducted with reference to this central frequency of the AOD and the ultrafast laser wavelength of 900 nm. Based on Eq. (2), the calculated spatial dispersion constant, *σ_AOD_* is 0.00771°/nm. By rotating the prism and thus defining the angle of incidence of the laser, the spatial dispersion induced by the AOD can be cancelled out based on the condition that [26]

$$\sigma_{AOD}+\sigma_{prism}=0$$ (3)

where *σ_prism_* is the negative spatial dispersion constant of prism. *σ_prism_* being [27]

$$\sigma_{prism}=\frac{d\beta }{dn}\cdot \frac{dn}{d\lambda }$$ (4)

where *β* is the exit angle of the laser from the prism, *n* is the refractive index of the prism and $\frac{dn}{d\lambda }$ is the prism chromatic dispersion. The detailed derivation of $\frac{d\beta }{dn}$ can be found in Ref [27]. At 900 nm, the corresponding values of *n* and $\frac{dn}{d\lambda }$ for SF11 glass are 1.760 and −0.427°/nm respectively. Here, in our prism-AOD compensation analysis, we consider only the magnitude of $\frac{dn}{d\lambda }$. At the given apex angle, α, of the prism, *i.e.* 60°, the progression of the spatial dispersion constant with increasing incident angle can be plotted to find the intersection point at which both the values of *σ_prism_* and *σ_AOD_* equate and thus, helping to establish the correct angle of incidence of the laser at the prism for beam shape recovery. In our instance, the optimum laser incident angle estimated is 55.6°.

To restore the initial pulse width of Ti:Sapphire laser after passing through the AOD, we can calculate the temporal dispersion constant, $\frac{\text{Δ}\tau }{\text{Δ}\lambda }$ and subsequently derive the negative GDD obtained from the prism-AOD pair with the equivalent magnitude to compensate for the positive GDD of the AOD. The temporal dispersion constant is given as [27,28]

$$\frac{\text{Δ}\tau }{\text{Δ}\lambda }=-\frac{\lambda Lf}{cV}\cdot \frac{d\beta }{dn}\cdot \frac{dn}{d\lambda }$$ (5)

where *L* is the distance between the SF11 prism and the AOD and *c* is the speed of light. Based on Eq. (5), the corresponding negative GDD introduced in this scheme can be expressed as [27]

$$GDD_{prism-AOD}=-\frac{\text{Δ}\tau }{\text{Δ}\lambda }\cdot \frac{\lambda^{2}}{2\pi c}$$ (6)

Therefore, with the optimal incident angle of the laser predetermined from our earlier analysis of the spatial beam compression, the change of the resultant negative GDD in response to the increase in the distance between the prism and the AOD can be investigated.

**Fig. 2:**
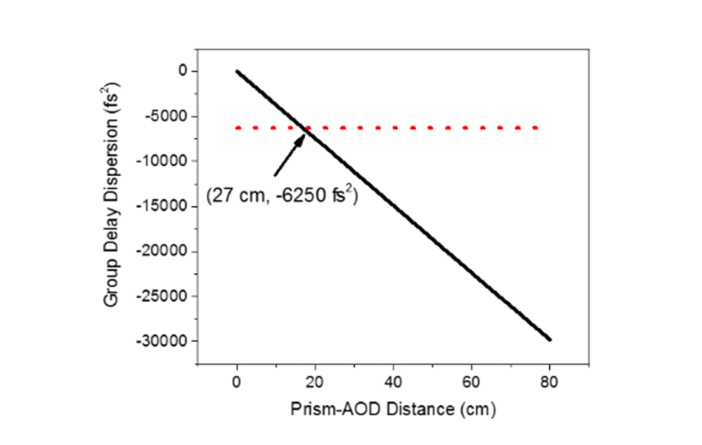
Calculated negative GDD provided by prism-AOD pre-compensation scheme as a function of spatial separation. The red dotted line shows the matching GDD value to that of material dispersion in AOD alone and thus, the intersection point is the predicted optimum distance between the prism and the AOD for restoring the pulse width of the laser tuned to 900 nm.

The TeO_2_ crystal in the AOD is highly anisotropic and specially cut to produce different pulse travel distances along the extraordinary axis and along the ordinary axis in the generation of first order beam diffraction [29]. As a result, at 900 nm, the effective positive GDD from the extraordinary and the ordinary axes was predicted to be 6250 fs^2^ [30]. This estimation was used as the preliminary guide to determine the separation of the AOD from the pre-compensation prism. At the distance, Eq. (6) produced the GDD with the equivalent value but the opposite sign to that of AOD. In this instance, at −6250 fs^2^, the corresponding prism-AOD distance was 27 cm, as shown in Fig. 2

. The optimum condition for the empirical temporal dispersion compensation at the excitation wavelength of 900 nm was later acquired by measuring the resulting pulse width stepwise along the prism-AOD track using an interferometric autocorrelator (pulseCheck, APE, Germany).

#### 2.1.2 Remote focusing for high numerical aperture axial switching

**Fig. 3:**
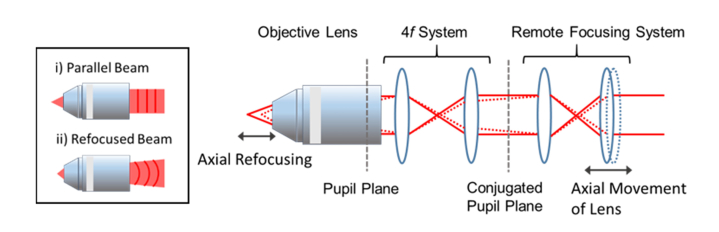
Schematic diagram of remote focusing configuration in which the back pupil plane of the objective is reimaged onto the pupil plane of the remote focusing lens by a 4*f* system. The translation of the rightmost lens in the refocusing system along the optical axis affords the axial scanning of the focal spot, without direct mechanical movement of the objective lens, as guided by the simple illustration of the marginal ray paths. Inset depicts the shape of the laser wavefront when collimated or refocused away from the nominal focal plane.

In this form of *in vivo* imaging, optical remote focusing offers the capability of fast switching between different focal planes as the mechanical translation of the objective lens or the specimen is near impossible at the required speed. Many microscopes used in chronic brain imaging employ a tiltable objective lens in order to image through a slanted cranial window that makes direct mechanical refocusing impractical. In view of that, we opted for the remote focusing technique to image the neuronal circuits inside the mouse brain at different depths without the direct mechanical disturbance of the objective lens or the animal [12,13,31]. Remote focusing is a method whereby the focus is translated in an aberration-free way along the optical axis. This is implemented by imaging the pupil of the second lens with matched NA onto the pupil of the objective lens, as illustrated in Fig. 3

. Thereby, when the rightmost lens in the depicted refocusing system is axially displaced, the changes in the wavefront, specifically the beam divergence or convergence, at the conjugated plane of the matching lens will be replicated but with the opposite sign at the pupil plane of the objective lens. Thisleads to the beam refocusing without introducing spherical aberration [12,32,33]. In other words, without any motion in the vicinity of the specimen, the focus can be translated axially over a large range without significant aberration. Note that this is only possible if the aperture angle of the objective and the refocusing system are matched; if lower NA lenses are used in the refocusing system, then significant spherical aberration will be introduced in addition to the beam refocusing. The reason for this is that the pupil phase introduced by low NA refocusing lenses is represented by a quadratic function of radius, while the refocusing phase for a high NA objective contains higher order terms [12,32]. The pupil phase of the high NA objective can be described as

$$\phi (\rho )=n_{objective}\cdot k\cdot z\cdot \left(1-s^{2}\frac{\rho^{2}}{2}-s^{4}\frac{\rho^{4}}{8}+\ldots \right)$$ (7)

where *n_objective_* is the refractive index of objective lens immersion medium, *k* is the wavenumber in free space, *z* is the axial focal displacement from the nominal plane, *s* is the sine of the objective half-angle and *ρ* is the normalised pupil radius. Therefore, the presence of the higher order terms when using a low NA lens to perform high NA refocusing means that refocusing occurs only with additional spherical aberration.

To implement axial refocusing at high NA without giving rise to considerable spherical aberration, the remote focusing lens chosen has to satisfy the sine and the Herschel conditions from which can be reduced to [12,32]

$$\theta_{objective}=\theta_{refocusing}$$ (8)

where *θ_objective_* is the semi-aperture acceptance angle of objective lens and *θ_refocusing_* is the semi-aperture acceptance angle of the remote focusing lens. Thus, Eq. (8) can be further expanded to

$$\frac{NA_{objective}}{NA_{refocusing}}=\frac{n_{objective}}{n_{refocusing}}$$ (9)

where *NA_objective_*, *NA_refocusing_* are the numerical apertures of the objective and the remote focusing lens respectively and *n_objective_*, *n_refocusing_* are the refractive indices of the immersion media for the objective and the refocusing lens. From that, the resulting axial focal displacement, *z,* in relation to the remote focusing lens translation as illustrated in Fig. 3, can be expressed as [12]

$$z=\frac{n_{refocusing}}{n_{objective}}\cdot Z_{refocusing}$$ (10)

where *Z_refocusing_* is the translation of the remote focusing lens.

The straightforward solution to the axial refocusing scheme is, of course, to construct a system with identical objective lenses – or at least those with matched aperture angles – where the condition set by Eq. (8) will be satisfied automatically. However, especially for *in vivo* multiphoton imaging, a reasonably high NA (NA > 0.6) focusing objective is generally utilised to create the high photon flux at the focal volume for non-linear excitation of the fluorescent markers. The conventional solution to this would be to use a matched objective lens in a reflection mode with an axial scanning mirror or to use two face-to-face objective lenses in a transmission configuration. Although water dipping lenses are commonly used in brain imaging in order to provide large working distance and reduced refractive index mismatch, the focusing lenses can be without immersion, as long as the aperture angles are matched. Here, we chose to use high NA aspheric lenses designed to correct for the spherical aberration for on-axis focusing as an alternative replacement for the objective lenses in the remote focusing system [14]. This approach enables high NA refocusing at an appreciably lower cost than using conventional microscope objective lenses. We note that although the aspheric lenses are only corrected for aberrations on-axis, whereas objective lenses also have off-axis correction, only on-axis performance is required if the focusing system is placed before the scan mirrors. In accordance to Eq. (9), the free space aspheric lenses should have NA of 0.60 or higher given that the water dipping objective (CFI75 LWD 16 × W, Nikon, Japan) used for the excitation here has NA of 0.8. On that basis, off-the-shelf aspheric lenses of 0.70 NA (C330TMD-B, Thorlabs, USA) were acquired for the remote focusing system.

#### 2.1.3 Spatial and temporal compensation for acousto-optic deflector induced dispersion

We calculated the incident angle of the laser at the prism and its distance from the AOD required to optimally compensate for the spatial and temporal dispersion of the AOD. Given the central acoustic frequency of 87.5 MHz for the ultrafast laser wavelength 900 nm, we arrived at the estimated values of 55.6° and 27 cm respectively. These were further fine-tuned experimentally in the process of constructing the prism-AOD pre-compensation unit. While varying the angle of incidence of the laser at the prism between 55° and 57°, hence changing the spatial dispersion constant *σ_prism_*, the intensity profile of the laser exiting the prism-AOD unit was monitored with the beam focused on a CMOS sensor (LifeCam, Microsoft, USA) using an achromatic doublet with the focal length of 60 mm. The distance between the prism and the AOD was kept momentarily at ~22 cm.

**Fig. 4:**
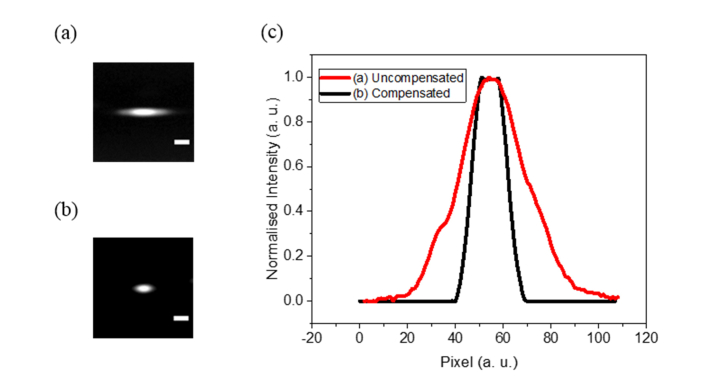
Far field beam profiling in uncompensated and spatial dispersion compensated AOD settings. (a) Uncompensated laser beam spot focused by achromatic doublet with 60 mm focal length. (b) AOD dispersion compensated laser focal spot. (c) Comparison between uncompensated and compensated beam intensity lineshapes. Scale bar represents 50 µm.

In comparison with the uncompensated beam propagating through the AOD, the broadened beam waist was found to be markedly reduced by a factor of ~2 when the incident angle of the laser was tuned to ~56° (Fig. 4(c)

). This is in good agreement with the predicted value in restoring the laser beam shape.

**Fig. 5:**
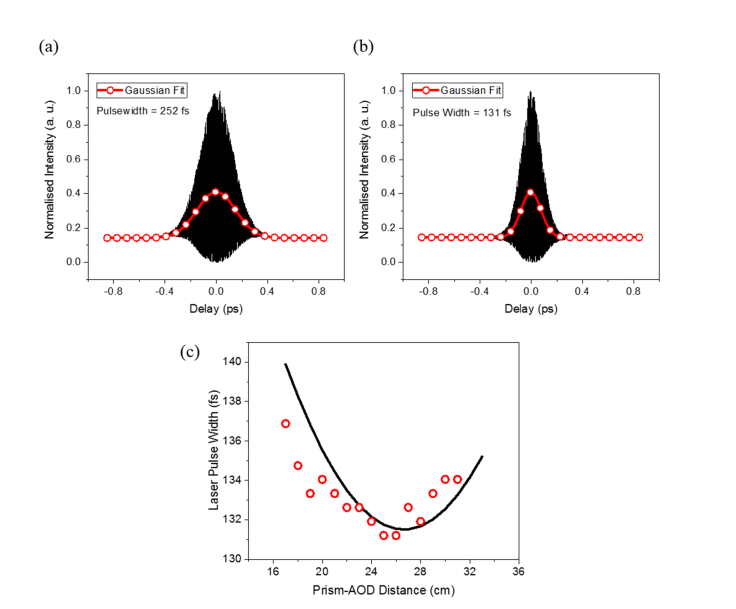
Pulse width measurements of diffracted laser beam at varying degree of temporal dispersion compensation. (a) Measured interferometric autocorrelation (IAC) trace with fitted Gaussian function for the laser beam without dispersion compensation. (b) IAC trace and associated Gaussian fit for compensated laser beam at minimum temporal dispersion. The laser pulse duration was calculated from dividing full-width-at-half-maximum (FWHM) of IAC by a factor of 1.41 [34]. (c) Progression of measured laser pulse width as a function of prism-AOD distance.

Following the fine tuning of the angle between the normal of the prism and the incident laser in achieving the optimum spatial dispersion correction, the output pulse width was examined using the interferometric autocorrelator as the prism-AOD distance was increased stepwise. The prism-AOD spatial separation was roughly estimated from a ruler and the corresponding autocorrelation interferogram was recorded at 1 cm intervals starting from ~17 to ~32 cm. Assuming the ultrafast laser pulse shape followed the Gaussian envelope, the pulse duration was extracted by applying 0.71 factor to the FWHM of the interferogram. From the series of autocorrelator measurements, the shortest pulse width of 131 fs was attained when the prism and the AOD were separated by ~25 cm. Compared to the uncompensated pulse width of 252 fs, we achieved a reasonable pulse compression by a factor of ~2. However, there was a slight discrepancy between the initial pulse duration measured, *i.e.* 107 fs, at the laser output, and the minimal pulse width acquired via prism-AOD pre-compensation configuration. The difference could be due to the residual material GDD of the dispersive SF11 prism. To check the dependency of the temporal compensation on the prism-AOD separation, the experimental pulse width measurements were fitted with Eq. (11) as a function of prism-AOD distance [29,35,36],

$$\tau_{out}=\tau_{in}\cdot \sqrt{1+{\frac{\left(4\ln2\cdot (GDD_{AOD}+GDD_{prism-AOD})\right)}{{\tau_{in}}^{4}}}^{2}}$$ (11)

where *τ_out_* is the output pulse width after the prism-AOD, *τ_i_*_n_ is the input pulse width, *GDD_AOD_* is the positive GDD of the AOD and *GDD_prism-AOD_* is the resultant negative GDD generated by the prism-AOD pre-compensation unit with incremental *L* distance as expressed by Eq. (6). On the basis that the value of *GDD_AOD_* parameter was 6250 fs^2^ as provided by the manufacturer and the *τ_in_* value was fixed at 131.5 fs, the final output pulse duration was calculated. In this instance, we momentarily discounted the difference in the pulse width between the initial and the optimal pre-compensation conditions as it did not contribute to the variation in the final pulse width with the increment in the prism-AOD distance. As depicted in Fig. 5(c)

, the change in the pulse duration with increasing prism-AOD distance as observed experimentally is in good agreement with the predicted trend, albeit there is a small offset between the trough positions at which the minimum pulse width was measured. This is because it can be difficult, in practice, to precisely measure the actual distance between the prism and the AOD.

## 3. Hardware development and experimental methods

### 3.1 Quasi-simultaneous multiplane microscopy construction

**Fig. 6:**
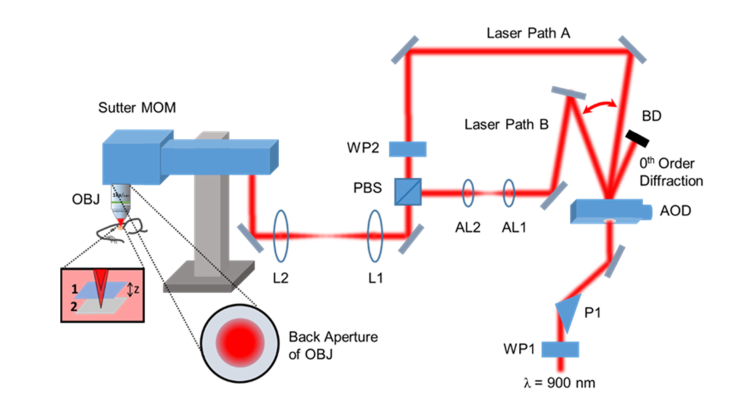
Simplified schematic diagram of multiplane functional imaging system. MOM: Moveable Objective Microscope with closely coupled green and red fluorescence detectors; WP1, WP2: half-wave plates; P1: SF11 equilateral prism; AOD: acousto-optic deflector; BD: beam dump; AL1, AL2: aspheric lenses; PBS: polarising beamsplitter; L1, L2: achromatic doublets; OBJ: objective lens. Insets depict the optical focal switch between two planes separated by z distance and the partially-filled back aperture of excitation objective lens. Laser Path A represents the nominal in-focus beam path and Laser Path B represents the refocusing beam path.

The quasi-simultaneous multiplane functional imaging system was built around a Sutter Moveable Objective Microscope (MOM, Sutter, USA) that offers a slimline microscope frame with accessible window to the resonant scanner, which allows easy hardware integration for additional bespoke imaging modalities (see Fig. 6

). A femtosecond Ti:Sapphire laser (Mai Tai BB, Spectra-Physics, USA) tuned to 900 nm was used as the two-photon excitation source. The components that made up the add-on multiplane functional imaging modality primarily consisted of the DTSX-400-980 AOD for fast inertia-free optical switching, the SF11 equilateral prism for pre-compensation of AOD material dispersion and the C330TMD-B aspheric lenses for high NA remote focusing. The normal of the prism was fixed at ~56° with respect to the incident excitation laser at which angle the focused beam spot was detected to be the smallest using the CMOS camera. The prism was placed ~25 cm prior to the AOD at which distance the resultant pulse width after propagating through the AOD was measured to be the shortest using a collinear autocorrelator. The remote focusing system comprised two face-to-face C330TMD-B aspheric lenses with the first lens, AL1 mounted on a 3-axis fine translation stage (MBT616D/M, Thorlabs, USA) while the second lens, AL2 was fixed in position. The acoustic frequency of the AOD was programmed to switch between 87.5 ± 4 MHz, *i.e.* 83.5 MHz and 91.5 MHz respectively, to provide two distinct optical paths: (1) one bypassed the remote focusing system leading to the nominal focus; (2) the other encompassed the refocusing system leading to the axial focal displacement. Both beam paths were then recombined with a polarising beamsplitter. The AOD access time in the microsecond range permitted near simultaneous imaging of two focal planes at different depths *in vivo*. A cascade of 4*f* telescopic imaging systems were constructed utilising pairs of achromatic doublets so as to reimage the pupil plane of the aspheric lens onto the objective lens. Given that *in vivo* imaging generally suffers some degree of specimen motion, especially in the axial direction, it is desirable to have an extension of the depth of focus so that the neurons of interest stay in the image, even in the presence of specimen movements. For this reason, the beam was expanded to partially fill the back aperture of the objective lens to about 50% of the aperture diameter. This created an extended axial focus with an approximate length of 12 µm. The emitted fluorescence was captured by the full NA of the objective lens, thus maintaining the detection efficiency. This configuration pre-emptively makes the multiplane functional imaging more robust to the specimen motion, maintaining the focus on the active neurons during imaging over a period of time and thus allowing the continual recording of neuronal firing patterns inside the mouse brain. The image acquisition was performed using ScanImage software (Vidrio Technologies, USA) with some minor modifications for the AOD beam steering control. For simplicity, the AOD switching occurred at every frame acquisition, though other switching modes like alternating line scans would also be possible owing to the short AOD access time. The highest achievable frame rates for both the in-focus and refocused planes were 15 fps at 512 × 512 pixels; in turn, we could perform video rate imaging on each plane at reduced image pixel number *i.e.* 256 × 256.

### 3.2 Transgenic mouse surgical procedures and multiplane calcium imaging

All animal procedures were approved by the local ethical review committee and performed in accordance with Home Office regulations. We used one adult (postnatal day 45) male mouse constitutively expressing the fluorescent calcium indicator GCaMP6s in excitatory neurons (offspring from a cross between Ai95(RCL-GCaMP6s)-D and T29-1.CaMKIIalpha-cre mice, The Jackson Laboratory (Bar Harbor, USA) stock numbers 024105 and 005359 respectively [37,38]). General anaesthesia was induced by administering 5% isoflurane in O_2_ at 1 L/min for 30 seconds. The mouse was then held in a stereotaxic apparatus and anaesthetised throughout the procedure by delivering 1.5 ± 0.5% isoflurane in O_2_ at 0.2 L/min. Additional drugs provided analgesia (Vetergesic (Ceva Animal Health, UK) 0.1 mg/kg, intraperitoneal; Marcain Polyamp (AstraZeneca, UK) 2 mg/kg, subcutaneous under scalp) and anti-inflammatory effects (Metacam (Boehringer Ingelheim, Germany) 5 mg/kg, intraperitoneal). Eye cream (Lacri-Lube, Allergan, UK) was applied to the eyes. Body temperature was monitored and maintained at 36 ± 1 °C. A circular piece of scalp was removed and the underlying skull was cleaned and dried. A circular craniotomy (3 mm diameter) was centred over the right somatosensory cortex of the mouse located using stereotaxic coordinates. The craniotomy was sealed with a glass cover slip and cyanoacrylic glue (Loctite, Germany). A custom-made stainless steel head fixation plate was cemented onto the skull using dental cement (Super-Bond C&B, Sun Medical, Japan). Two weeks after the surgery, the mouse was habituated to handling and gentle restraint over two to three days, before being head fixed and trained to run on a styrofoam cylinder (22 cm diameter, on a ball-bearing mounted axis) in the dark over the course of four days. Three weeks after the surgery, two-photon calcium imaging (excitation at 900 nm with the frame rate of 15 fps at the in-focus and refocused planes) was performed during head fixation on the running wheel cylinder. A metallic pole attached to a stepper motor was made to contact the whiskers every 5 seconds.

### 3.3 Image analysis

Mechanical drift in the x and y planes was corrected offline using the Suite2p analysis package [39]. Suite2p was also used to identify the regions of interest (ROIs) corresponding to neuronal cell bodies. A separate time-averaged image was then generated from all recorded frames in a given field of view. For each neuron, the signal at each time frame was calculated as the average fluorescence, F(t), across all pixels inside the ROI. The baseline fluorescence, F_0_, for a given ROI was calculated as the median of the 10th to 70th percentile of fluorescence values across all frames. The fluorescence time series for the neuron was then corrected for the baseline using the formula, (F(t) – F_0_)/F_0_, which is commonly denoted as ΔF/F_0_.

## 4. Experimental results and discussion

### 4.1 Remote focusing point spread function measurements

Because, in mouse, different neocortical layers are separated by at least 100 µm, any multiplane imaging methods developed should ideally cover focal planes that axially are separated by many hundreds of micrometres. Therefore, we investigated, within the axial scan range of ~400 µm, the intensity profile of the axial point spread function (PSF) and the relative 2P excitation efficiency of the remote focusing system. This was performed on a 3D fluorescent bead sample (0.5 µm yellow-green fluorescent beads (Polysciences, USA) embedded in 10% (w/v) agarose gel) at a constant excitation power of ~60 mW, implemented through the translation of one of the 0.70 NA aspheric lenses. Here, the relative 2P excitation efficiency is expressed in terms of the fluorescence intensity, I_PSF_ where the total intensity collected over the excited 0.5 µm fluorescent bead is divided by the axial FWHM of the PSF (n = 10 samples). The final mean values were then normalised to that of the fluorescence intensity at the zero refocusing setting.

**Fig. 7:**
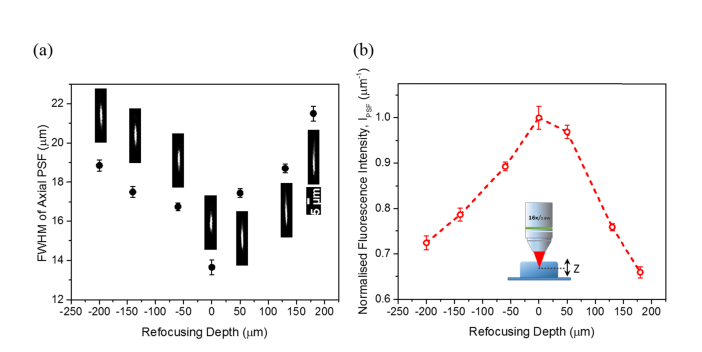
Investigation of two-photon excitation from the remote focusing. (a) FWHM of axial point spread function at varying refocusing depths derived from imaging 0.5 µm yellow-green fluorescent beads embedded in 10% (w/v) agarose gel. (b) Measured fluorescence intensity, that I_PSF_ is the sum of PSF intensity over axial FWHM, at different refocusing depths. Error bars shown in both figures represent the standard error of the mean.

We observed no marked differences in the lateral resolution – the value remained largely around ~1.6 µm across various scan depths. Compared to the theoretical estimation of ~1.1 µm for the half-filled objective lens, the lateral resolution determined empirically appears to be lower. From Fig. 7(a)

, it can be seen that the axial size of the PSF is minimum, *i.e.* ~13.7 µm, at the zero refocusing and starts to broaden as the displacement of the focal spot from the nominal focal plane increases. The initial axial resolution measured at the zero refocusing is slightly lower than the predicted with the value of ~12.5 µm. These experimental observations can be attributed to the incomplete cancellation of the higher order phase terms at the objective pupil plane by the less perfect reimaging condition between the high NA aspheric singlet lens and the water dipping compound objective lens, added to the possible presence of the residual spatial dispersion from the AOD. Therefore, the wavefront distortion, predominantly the longitudinal spherical aberration, further manifested itself as the spread of the axial intensity distribution of the PSF, when the distance between the aspheric lenses in the remote focusing system is either increased or decreased to shift the focal spot away from the nominal plane. Apart from that, the scattering effect in the 3D bead sample can also lead to the elongation of the axial PSF especially when the excitation beam is refocused deeper into the sample and thus, contributing to the loss of symmetry in the excitation efficacy over the refocusing depth as illustrated in Fig. 7(b). As we intended to create images with an extended depth of focus, these extra PSF broadening effects were not considered to be detrimental to the imaging task.

### 4.2 In vivo quasi-simultaneous multiplane calcium imaging of mouse somatosensory cortex

Here, we demonstrated the usefulness of the quasi-simultaneous multiplane imaging technique we developed for fast recording of neuronal activity in two separate neocortical layers.

**Fig. 8:**
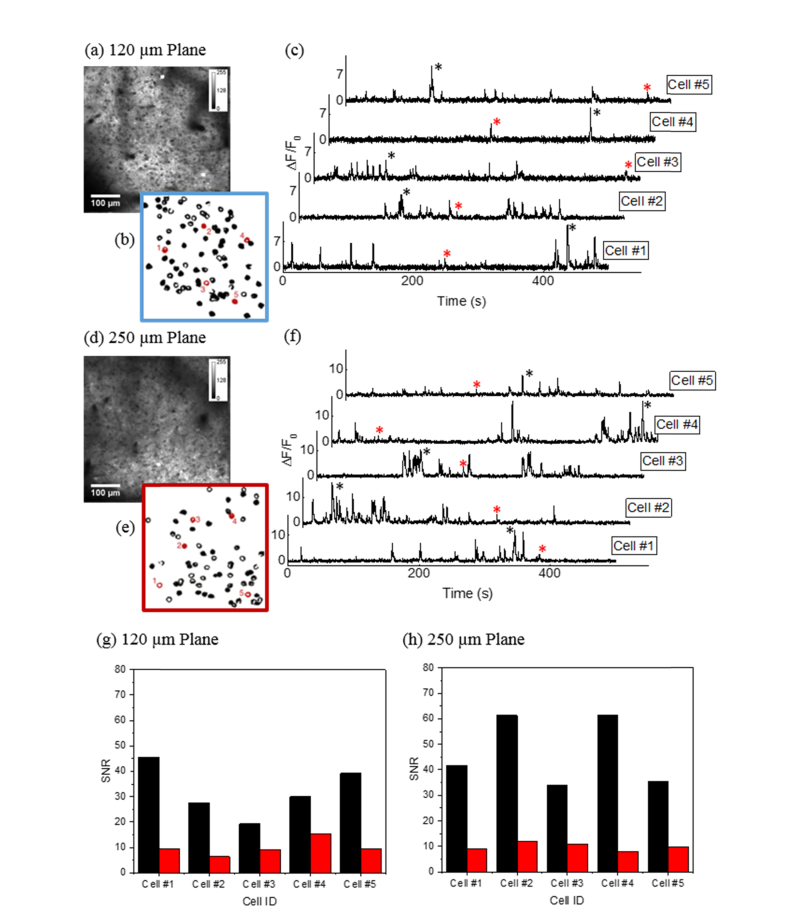
*In vivo* multiplane calcium imaging of neuronal circuit expressing GCaMP6s in the mouse parietal cortex region. Time-averaged fluorescence images of neurons at (a) the in-focus plane, 120 µm below the brain surface and (d) the refocused plane, 250 µm below brain surface. (b), (e) Regions of interest corresponding to the neurons in (a) and (d) with the location of five representative cells highlighted in red. (c), (f) Representative fluorescence traces from the five marked neurons at the in-focus and the refocused planes. (g), (h) Signal-to-noise ratio derived from the fluorescence transients at the in-focus and the refocused planes with the corresponding peaks labelled with asterisk sign (*).

The nominal in-focus plane was 120 μm below the brain surface, while the refocused plane was 250 μm below the brain surface (Fig. 8(a), (d)

). The amplitude modulation for the AOD was typically tuned to 2 V for the in-focus plane and 3 V for the refocused plane, corresponding to an average excitation power of ~80 mW and ~140 mW respectively. Time-lapse fluorescence images from individual focal planes (500 × 500 μm) in the parietal cortex of a head-fixed mouse running on a wheel were acquired to extract the fluorescence transients. The fluorescent calcium indicator GCaMP6s provides an indirect measure of neuronal activity where the active neurons within the field of view were identified as ROIs (Fig. 8(b), (e)). Neuronal activities are represented by the fluorescence fluctuations from each ROI over a period of time (Fig. 8(c), (f)). It is clear from the graphs of ΔF/F_0_ that the calcium signals are discernible over the baseline in both the in-focus and refocused planes. In order to quantify the signal-to-noise ratio (SNR), we chose, as exemplars, two peaks from each trace, *i.e.* one larger and one smaller peaks, and calculated the corresponding SNRs as shown in Fig. 8(g) and (h). The SNR was derived by dividing the peak amplitude of ΔF/F_0_ by the standard deviation of the baseline signal during a two second period. For the smaller peaks, the SNRs are in excess of 5, while that for many of the larger peaks are an order of magnitude higher. This clearly demonstrates that the technique is capable of capturing neuronal signalling from imaging planes separated by more than 100 µm in depth at high imaging speed and contrast.

## 5. Conclusion

**Fig. 9:**
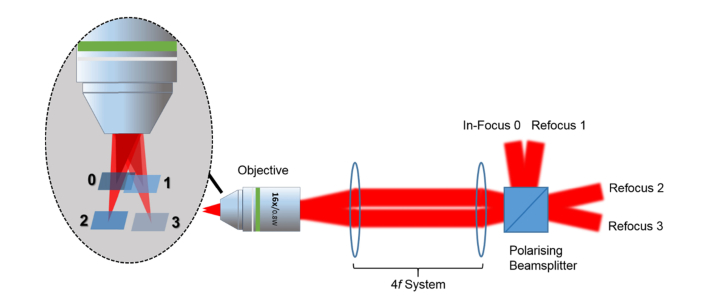
Simplified schematic diagram of expanded concept of quasi-simultaneous multiplane multiphoton imaging technique using AOD optical switching and remote focusing. The frame rate will be ~7 fps at respective focal planes.

We have developed a functional imaging technique that allows deep *in vivo* imaging of neuronal circuits at different focal planes quasi-simultaneously without loss of lateral resolution via the combination of remote focusing and AOD optical switching techniques. We have demonstrated the practicality and usefulness of the method in performing brain imaging on a transgenic mouse to extract the neuronal firing patterns in two different cortical layers. The cost of the implementation is significantly lower compared to a NA matched objective lens based refocusing system and can be easily integrated into existing microscopy systems from both hardware and software perspectives. The concept of using acousto-optic switching between different remote focused beam paths could be extended to more planes, beyond the two demonstrated, at reduced frame rate using a combination of spatial and polarization beam combination as depicted in Fig. 9

.
